# Acousto-optic holography for pseudo-two-dimensional dynamic light patterning

**DOI:** 10.1063/5.0185857

**Published:** 2024-04-03

**Authors:** Walther Akemann, Laurent Bourdieu

**Affiliations:** 1 Institut de Biologie de l’ENS (IBENS), École Normale Supérieure, CNRS, INSERM, Université PSL, 75005 Paris, France; 2 Laboratoire Kastler-Brossel (LKB), École Normale Supérieure, CNRS, Sorbonne Université, 75005 Paris, France

## Abstract

Optical systems use acousto-optic deflectors (AODs) mostly for fast angular scanning and spectral filtering of laser beams. However, AODs may transform laser light in much broader ways. When time-locked to the pulsing of low repetition rate laser amplifiers, AODs permit the holographic reconstruction of 1D and pseudo-two-dimensional (ps2D) intensity objects of rectangular shape by controlling the amplitude and phase of the light field at high (20–200 kHz) rates for microscopic light patterning. Using iterative Fourier transformations (IFTs), we searched for AOD-compatible holograms to reconstruct the given ps2D target patterns through either phase-only or complex light field modulation. We previously showed that phase-only holograms can adequately render grid-like patterns of diffraction-limited points with non-overlapping diffraction orders, while side lobes to the target pattern can be cured with an apodization mask. Dense target patterns, in contrast, are typically encumbered by apodization-resistant speckle noise. Here, we show the denoised rendering of dense ps2D objects by complex acousto-optic holograms deriving from simultaneous optimization of the amplitude and phase of the light field. Target patterns lacking ps2D symmetry, although not translatable into single holograms, were accessed by serial holography based on a segregation into ps2D-compatible components. The holograms retrieved under different regularizations were experimentally validated in an AOD random-access microscope. IFT regularizations characterized in this work extend the versatility of acousto-optic holography for fast dynamic light patterning.

## INTRODUCTION

Optical holography, in general terms, uses the spatial amplitude and phase distribution of electromagnetic light fields to encode information in the spatial domain.^1^ In this process, information is translated into visually perceivable virtual objects or, in other words, spatial structures made of light. Offering a large bandwidth and throughput, holographic light patterning has many applications, including use for data display, data storage, 3D imaging, lithography, additive manufacturing, cryptography, optogenetics, remote sensing, and the visual arts.^2–12^ Data coding through modulation of the optical phase rather than intensity fosters in-to-output efficiency, while digital generation of holograms using classical electrodynamic theory^13^ helps integration of optical and digital operations.^14^

On the practical side, dynamic holography typically needs to balance the temporal and spatial resolution available from spatial light modulator (SLM) devices. Nematic liquid–crystal SLMs provide a high pixel resolution (1–5 MP; mega pixel) for phase modulation at a moderate cycle time (20–100 Hz, with custom-designed devices of up to 500 Hz^15^), whereas micro-electromechanical system (MEMS)-based movable mirrors have fast cycle times (10–200 kHz) at a modest pixel resolution (0.1–4 kP; kilo pixel). Acousto-optic deflector (AOD) devices are less employed, despite their fast cycle times (20–200 kHz) as the modulation by a single AOD is limited to one dimension at an intermediate line resolution (0.05–0.4 kP). Acousto-optic (AO) light modulation, on the other hand, excels if speed is crucial and modulation in a restricted geometry is adequate. One example is low-noise holographic image projection, where 2D image frames are reconstructed from sets of 1D acousto-optic line holograms through a line-by-line sequential projection.^16^ Another example is ps2D acousto-optic light patterning for 3D targeted fluorescence excitation in recordings of physiological activity in *in vivo* nervous systems, for instance the mouse brain,^17^ or feedback-controlled adaptive correction of light scattering.^18^

An intriguing aspect of AO devices, next to their speed, is their capacity to generate complex holograms through simultaneous frequency (FM) and amplitude (AM) modulation of the AOD acoustic carrier wave.^17,19^ Complex holograms are recognized for superior reconstructions of holographic objects by reproducing the integral amplitude and phase structure of the light field.^20,21^ At the same time, they are difficult to generate with the current state-of-the-art 2D modulators, which only access the phase.^22–25^ AOD systems could provide a robust implementation of complex holography in a restricted geometry, while also offering a unique opportunity to compare the outcomes of phase-only (FM) vs complex (FM/AM) holography in one and the same device. However, AO holograms need to account for the operational properties of AODs, very different from standard SLMs. Yet, adapted methods to retrieve computer-generated holograms (CGHs) fitting AO modulators are missing. To fill the gap, we present a computational framework to obtain suitable acousto-optic holograms for microscopic light patterning. By accounting for AOD-specific constraints, we tested several regularizations of the Gerchberg–Saxton algorithm^26^ for the retrieval of either phase-only or complex AO-CGHs in pseudo-2D (ps2D) geometry and evaluated the outcomes with regard to reconstruction fidelity, noise rejection, and power budget.

In the following, we first detail the methodology for creating AO ps2D phase holograms [referred to as ps2D phase iterative Fourier transformations (IFT) and ps2D apodization IFT] used in Ref. 17 to tailor sparse grid patterns that enable fast 3D functional imaging at kHz sampling rate in awake mice. As these algorithms only permit for the reconstructions of sparse target patterns, but fail in the case of dense ps2D target objects because of intensity speckle noise, we then introduce a new algorithm for AO hologram retrieval in the FM/AM mode, ps2D complex IFT, supporting speckle-free rendering of dense ps2D objects. In particular, we introduce a way to efficiently account for the presence of a circular system aperture while generating ps2D target objects, which also improves ps2D phase IFT and ps2D apodization IFT. Finally, to overcome the geometrical constraints due to the ps2D geometry, we introduce fast sequential generation of ps2D patterns to create, at a still high temporal resolution, ps2D-unconstrained patterns.

## BACKGROUND: ACOUSTO-OPTIC SPATIAL LIGHT MODULATION

**FIG. 1.**
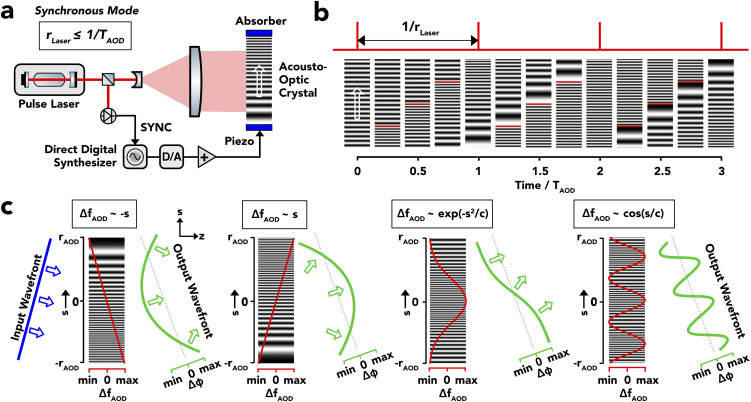
Acousto-optic wavefront shaping of low-repetition laser pulses. (a) Time-locked generation of acousto-optic phase gratings for diffraction of femtosecond laser pulses by traveling ultrasound acoustic waves in an optical crystal under the temporal control of a laser emission clock (SYNC) with the goal to present an individually prepared diffraction pattern to every laser pulse. In this synchronous mode, the maximally usable laser rate, *r*_*Laser*_, is defined by the intrinsic cycle time of the AOD, T_AOD,_ by *r*_*Laser*_ ≤ 1/*T*_*AOD*_. (b) Illustration of the temporal relationship between the laser pulsing (*top*; red) and the optical density wave filling the AOD aperture at different instants (*below*; black and white) during the presentation of a sequence of diffraction patterns switching between the following (*left to right*): a carrier frequency wave, a wave with positive frequency chirp, a wave with positive frequency offset, and a wave with negative frequency chirp. The vertical red bar indicates the transition from the preceding to the current pattern. (c) Illustration of the transformation of an incoming planar light wave (blue) into a spatially modulated non-planar output wave (green) by Bragg diffraction from an acoustic density wave with Δ*f*_*AOD*_, the acoustic modulation envelope (red). *Left to right:* a negative linear chirp, a positive linear chirp, a Gaussian chirp, and a sinusoidal chirp. The output waveforms created (green) are convergent parabolic, divergent parabolic, error function-like phase step, and sinusoidal, respectively.

In AODs, a collimated laser beam is Bragg-diffracted [supplementary material Fig. 1(a)] from a traveling longitudinal or shear density wave^27^ in an optical crystal^28^ with the density wave carried by acoustic long-wavelength phonons close to the center of the crystal first Brillouin zone.^29^ The devices operate in a strictly serial mode. An externally supplied radio signal is transduced into a propagating ultrasound wave through a piezoelectric element attached to one face of the crystal, which, in turn, establishes a spatial phase grating across the crystal as a retarded spatial replica of the time-dependent input signal.^30^ By exploitation of a broad angular phase match in biaxial chalcogenide crystals,^31^ such as TeO_2_, AODs may reach 60%–80% first-order diffraction efficiency within a 10–50 mrad angular deflection range.^28,32^ Their intrinsic cycle time equals the time for an acoustic signal to sweep the aperture of the device and, therefore, depends on the aperture diameter (4–15 mm typically). Given the high phase velocity of acoustic sheer waves in biaxial crystals (650 m/s in TeO_2_),^28^ AOD cycle rates vary between 40 and 170 kHz. The principal of an optical density grating in a constant sweeping motion, however, creates an intrinsic time dependence of Bragg diffraction from frequency-modulated acoustic carriers. An AOD lens,^33,34^ for instance, uses linear frequency chirp to focalize light [supplementary material Fig. 1(b)]. However, in the case of (quasi-) continuous input light, the focus will move at the speed and in the direction of the diffracting acoustic wave [supplementary material Fig. 1(c)]. The leading method to stabilize the focus, indispensable to 3D random access focus control, consists of passing the beam through a second AOD fed with the same acoustic signal but in the counter direction.^34–36^ This stabilization, however, breaks down periodically when the limits of acoustic bandwidth are reached [supplementary material Fig. 1(d)], as well as for non-linear frequency chirp. The time dependence of the AOD optical transfer function has, for a long time, impeded the widespread use of AODs in holography. The situation has changed recently following the introduction of the synchronous AOD mode.^30,37^ In this mode, the generation of the acoustic modulation signal is time-locked to a pulsed laser source [Fig. 1(a)] in order to present every pulse with a complete and *de novo* established diffraction pattern, while blanking out intermediates [Fig. 1b]. While the synchronous mode limits the usable laser pulse rate to the AOD cycle rate [Figs. 1(a) and 1(b)], the rates are compatible with the refractory times of regenerative laser amplifiers.^30^ By eliminating all time-dependent terms, the AOD transfer function simplifies to a linear relationship between the local curvature of the output wavefront, *dφ*/*ds*, and the local acoustic frequency, Δ*f*_*AOD*_, measured as the offset of the local frequency from the global carrier frequency, namely, d*φ*/d*s* (*s*) = 2*π*/*v*_*ac*_ · Δ*f*_*AOD*_(*s*) with *v*_*ac*_, the acoustic phase velocity and s, the spatial coordinate along the active AOD axis.^30^ Central to the acousto-optic holography, the relationship allows us to convert any unwrapped 1D optical phase function into an equivalent acoustic frequency signal [Fig. 1(c)]. In the following, therefore, when referring to the optical phase, we implicitly extend to the related acoustic frequency modulation envelope.

## RESULTS

### Phase holography of sparse 1D patterns

**FIG. 2.**
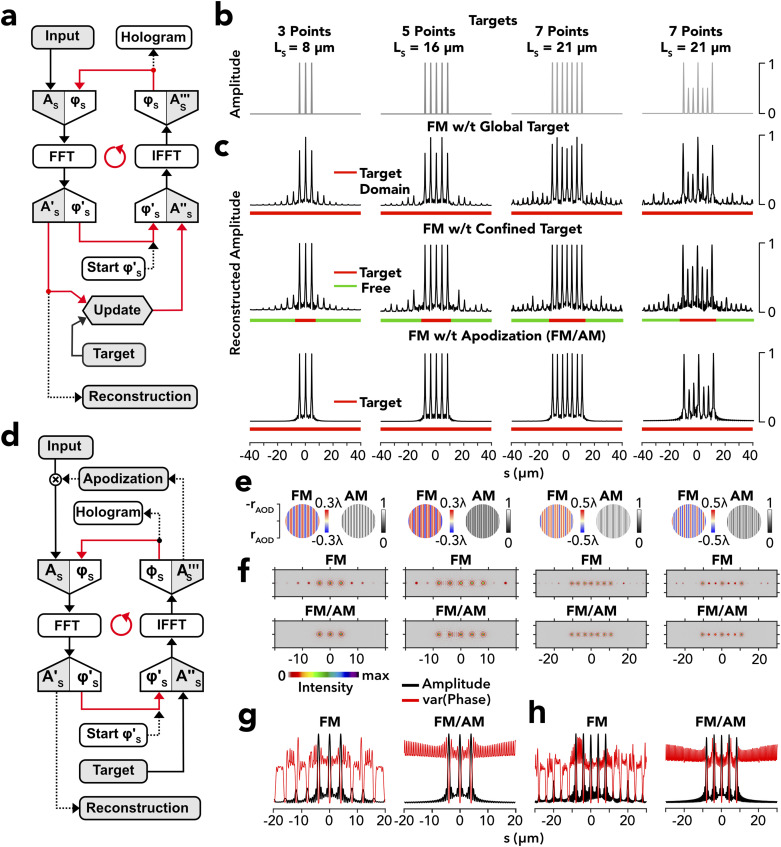
Phase holography for 1D discrete point patterns. (a) Flow diagram of the Gerchberg–Saxton algorithm for the retrieval of phase holograms from a given target pattern along a 1D Cartesian coordinate, s, by iterative Fourier transform (IFT) between the light fields in the target and holographic plane, with A, the amplitude, and φ, the phase. FFT and IFFT are the forward and inverse fast Fourier transformations, respectively. The iteration loop starts with a random phase in the target plane. During iteration, the target pattern is dynamically updated according to a fixed regularization rule (see Methods). The output is a phase hologram simply named “hologram.” (b) Example targets consisting of (from left to right) 3, 5, and 7 equidistant points of equal amplitude. The last target is identical to the seven-point target but with inhomogeneous amplitude. L_S_ denotes the overall target length. (c) Reconstructions obtained from the holographic output of IFT when imposing the targets in panel (b) using different algorithms. *Top row*: algorithm in panel (a) with a static target defined across the whole target plane. *Middle row*: algorithm in panel (a) with a dynamically updated target constraining only part of the target plane (target domain; horizontal red bar) with the complementary space unconstrained (freedom domain; green bar). *Bottom row*: algorithm in panel (d) with a static target across the whole target plane and apodization of the retained holographic phase. (d) Modified IFT for phase holography with apodization using a static target function. (e) False-color representation of the retained phase (blue to red; FM) and apodization amplitude (gray; AM) for the targets in (b) a 2D format. (f) 2D intensity reconstructions of the holographic phases in panel (e) in the FM mode with confined target (*top row*; FM) and FM/AM apodization mode (*bottom row*; FM/AM). The color code is given as the color bar. (g) Moving variance (square window 0.5 *µ*m) of the reconstructed phase (red) overlaid onto the reconstructed amplitude (black) of the 1D light field in the target plane for the three-point target [(c); *first column*] obtained without (*left*; FM) and with (*right*; FM/AM) amplitude apodization. (h) Same as panel (g), but for the five-point target [(c); second column].

Holographic image or pattern projection calls for a reverse-engineering of a hologram from a wanted intensity distribution in the front focal plane of an optical pattern, representing some kind of intensity object, yet without knowledge of the associated optical phase. Within the realm of paraxial Fourier optics,^38^ which may also extend to high numerical apertures,^39^ the problem is solved by the Gerchberg–Saxton algorithm,^26^ which exists in many variants^40–43^ and performs iterative Fourier transform (IFT) of the optical light field between the front and back focal planes of an optical system, in the following named target and holographic plane, respectively, by starting from a random phase in the target plane until self-consistency is reached [Fig. 2(a)]. To test different regularizations, we applied this algorithm [see Fig. 2(a) and Methods] to find phase holograms suitable to reconstruct simple line-ups of multiple discrete target points [Fig. 2(b)]. When imposing the targets as global functions across the entire target space, the reconstruction revealed inhomogeneous target peaks together with target side lobes (ghost spots) due to light diffracted into higher orders [Fig. 2(c), *upper row*]. The target error reflects the algorithm facing two incompatible objectives, namely, to render the wanted target and, to the same right, suppress target-associated diffraction lobes of higher order as the latter are clamped to zero in the target function supplied, thereby compelling IFT to minimize the global error across all diffraction orders.

A recognized strategy to avoid global over-regularization consists of segregating the target plane into a spatial domain hosting the target itself and a complementary freedom domain hosting the higher diffraction orders.^43,44^ In the freedom domain, the field is exempt from constrain and thus allowed to evolve freely during IFT.^45^ This results in a global target function being updated in each IFT cycle. We refer to this function as a dynamic target to differentiate from the fixed static target used before [Fig. 2(c), *upper row*]. Under dynamic regularization (see Methods), target peak amplitudes were well reconstructed, while target side lobes self-acquired full compatibility with the target without being set beforehand [Fig. 2(c), *middle* *row*].

While the side lobes are a fundamental consequence of light diffracted by a finite-sized aperture, they can be mediated by different processes, including contrast reduction of the phase hologram^46^ or weighted versions of the Gerchberg–Saxton algorithm, which control the amplitude of an input object during iterations.^47^ The side lobes are generally also amenable to amplitude tapering in the holographic plane.^48^ To test this second option, we reinstated the static target [as shown in Fig. 2(c), *upper row*] and applied the holographic amplitude, unused in the previous algorithm [Fig. 2(a)], as a post hoc apodization function to the final phase hologram found by IFT [Fig. 2(d)]. This so-called apodized hologram indeed yielded faithful reconstructions of the wanted targets in the absence of the target side lobes [Fig. 2(c), *bottom row*; *see also Fig. 2 in Ref. 17*]. In the view of Fourier optics, valid holograms emerge as a series of sinusoids with their Fourier spectrum approximating the target function. Therefore, discrete point targets [Fig. 2(b) and supplementary material Fig. 2(a)] are represented by harmonic phase and amplitude functions composed of two (equidistant three-point target), four (five-point), and six (seven-point) two-sided discrete frequencies. Because of the rectification that relates monophasic (amplitude) to biphasic (optical phase) signals, amplitude frequencies are non-linear harmonics of the phase comprising second harmonic [supplementary material Fig. 2(c)] as well as sum and difference spatial frequencies [supplementary material Fig. 2(d)]. For this reason, amplitude functions generally include high-frequency components transcending the phase. To adequately simulate the transformation of a light beam by an AOD device, we expanded the 1D holographic phase and amplitude functions to the whole device aperture [Fig. 2(e)] and calculated, by 2D Fourier transform, the resulting 2D light distribution in the image plane of an imaging objective [Fig. 2(f)] showing diffraction-limited intensity spots at intended x-axis positions [Fig. 2(b)]. To appreciate the effect of apodization, we assessed its effect on the phase distribution of the 1D- [Figs. 2(g) and 2(h)] and 2D-reconstructed light fields [supplementary material Figs. 3(a) and 3(b)]. In both cases, the target and side lobes coincide with the areas of constant phase or, equivalently, low phase variance [Figs. 2(g) and 2(h); *left*], while apodization resets the variance to the overall mean in the space not marked by the target points [Figs. 2(g) and 2(h); *right*]. In the example of sparse point targets, as investigated here, amplitude modulation thus turns out to create local phase variance to inhibit constructive beamlet interference outside the target peaks and, thereby, redistributes intensity to these targets. At the same time, apodization comes with the price of reduced power transmission. To elucidate this aspect, we varied the AM modulation gain between zero and one to evaluate the target error and power transmission for different gain settings using the five-point pattern as example [supplementary material Fig. 3(c)]. The target error monotonically decreased with a higher gain, from 17% at zero gain to almost zero at full gain [supplementary material Fig. 3(d)], in sync with a concomitant loss of power efficiency [supplementary material Fig. 3(e)].

### Complex holography of dense objects

Sparse targets are composed of discrete points with minimal overlap of their single-point diffraction lobes. On the other hand, if target points are placed densely enough for their Airy disks to overlap, components of the coherent light field will begin to interfere to produce high frequency phase fluctuation in the target plane, which, in turn, translates into amplitude noise, known as speckle noise, as typically appearing in phase-only reconstructions of diffusively illuminated dense objects.^49^ Partial denoising is achievable by domain mixing, originally conceived to denoise holographic optical tweezers,^40^ which relegates the noise from the object itself to the surrounding domain [supplementary material, Figs. 4(a)–4(e)]. The method, therefore, augments the noise outside the target, while reducing the power efficiency for the target itself [supplementary material, Fig. 4(c)].

**FIG. 3.**
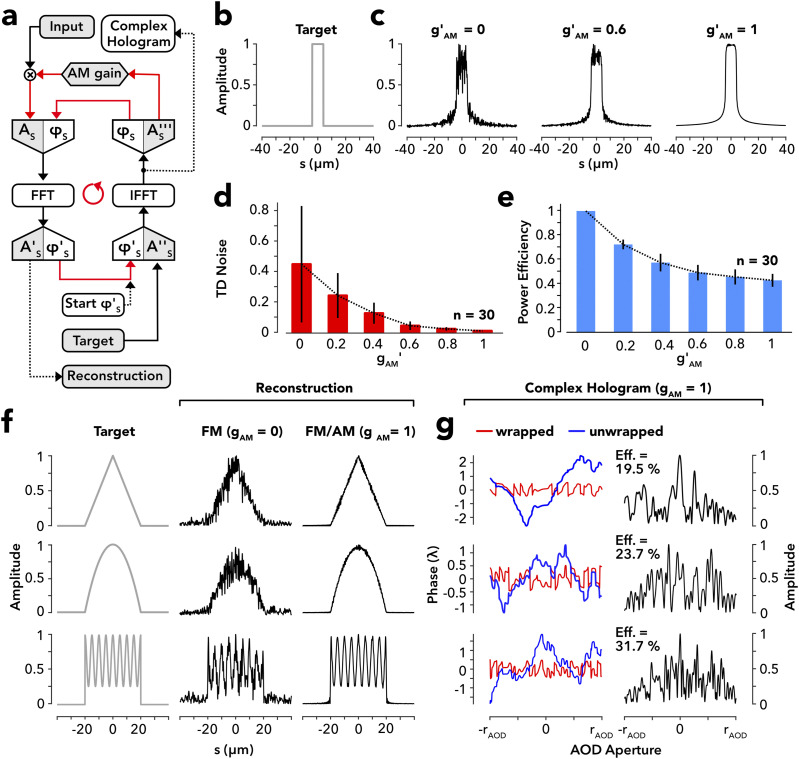
Complex holography of dense 1D targets. (a) Flow chart of the IFT algorithm, implementing combined iterative optimization of the holographic phase and amplitude by continuous amplitude feedback at variable gain. (b) Line segment (8 *µ*m) as an example of a dense target function. (c) Reconstructions of target (b) for different feedback gains. Amplitudes are normalized to one. The decadic gain, $g_{AM}^{\prime }\in \left[0,1\right]$, defined as $g_{AM}^{\prime }:=0.5\;log_{10}\left(1/\left(1-g_{AM}\right)\right)$ with *g*_*AM*_ ∈ [0, 0.99], the linear gain, is introduced for scaling purposes, *g*_*AM*_ refers to the maximum gain value reached at the end of the initialization ramp (see Methods). The suffix “max” is omitted to simplify the notation. (d) Speckle noise evaluated in the target domain, here defined as the coordinate range where the target function differs from zero, as a function of decadic gain, $g_{AM}^{\prime }$. Given are the means from 30 IFT repetitions (n = 30) with error bars representing the standard deviation. (e) Power efficiency of diffraction into the first Bragg order as a function of the decadic gain. The data represent the means and standard deviation. (f) A triangular, parabolic, and sinusoidal amplitude target (*left row*) with reconstructions at zero (*middle row*; *g*_*AM*_ = 0) and full amplitude feedback (*right row*; *g*_*AM*_ = 1). Amplitudes are normalized to one. (g) Retained holographic phase (red: wrapped; blue: unwrapped) and amplitude (black) representing the complex hologram for target patterns in panel (f) at full feedback (*g*_*AM*_ = 1).

In a potentially more powerful approach, denoising could instead aim to remove the very cause of the noise by providing the holographic field with the missing amplitude information. Amplitude apodization, however, has little effect on the on-target noise, despite mitigating the off-target noise [supplementary material, Figs. 4(f) and 4(g)], consistent with a repression of high-order intensity in the surrounding domain as seen before [Fig. 2(c)]. Therefore, apparently the apodization mask lacks the correct amplitude information relating to the on-target speckle structure. Indeed, the holographic field was optimized in complete disregard to the holographic amplitude [Fig. 2(d)]. We, therefore, introduced an additional loop into IFT to feed back the holographic amplitude to the input during iteration alongside the phase [Fig. 3(a)]. To avoid early IFT stagnation, the feedback was regulated by applying a dynamic gain starting with zero and gradually increasing to its final setting (see Methods). The IFT output is designated as “complex hologram” to mark the difference to the phase-only hologram obtained before [Figs. 2(a) and 2(d)]. Complex holograms undergo simultaneous and inter-related IFT optimization of their amplitude and phase component and as such are distinguished from phase-only holograms, regardless of whether apodization is applied or not. To test this approach, we searched complex CGHs to reconstruct a short continuous line segment [Fig. 3(b); same as in the supplementary material, Figs. 4(a) and 4(f)]. While the reconstructed line exhibited a high level of speckle noise at zero feedback, the noise decreased from non-zero feedback to completely vanishing across the entire space domain at full gain [Figs. 3(c) and 3(d)]. At the same time, the denoising involved only a relatively modest efficiency loss [40% efficiency at full gain; Fig. 3(e)]. In this case, the loss is not by intensity redistribution within the target plane but transfer to an external power sink, which is the zero Bragg order in the case of the AODs. We confirmed these findings for different objects of triangular, parabolic, or sinusoidal shape, all yielding close to noise-free reconstructions at full gain [Fig. 3(f)]. The associated CGHs revealed a riche structure and large modulation depth [Fig. 3(g)], different from the point targets analyzed before [supplementary material Fig. 2(b)]. Together, the results suggest simultaneous optimization of the amplitude and phase as a means for speckle denoising in acousto-optic complex holography.

### From 1D to ps2D to serial holography

**FIG. 4.**
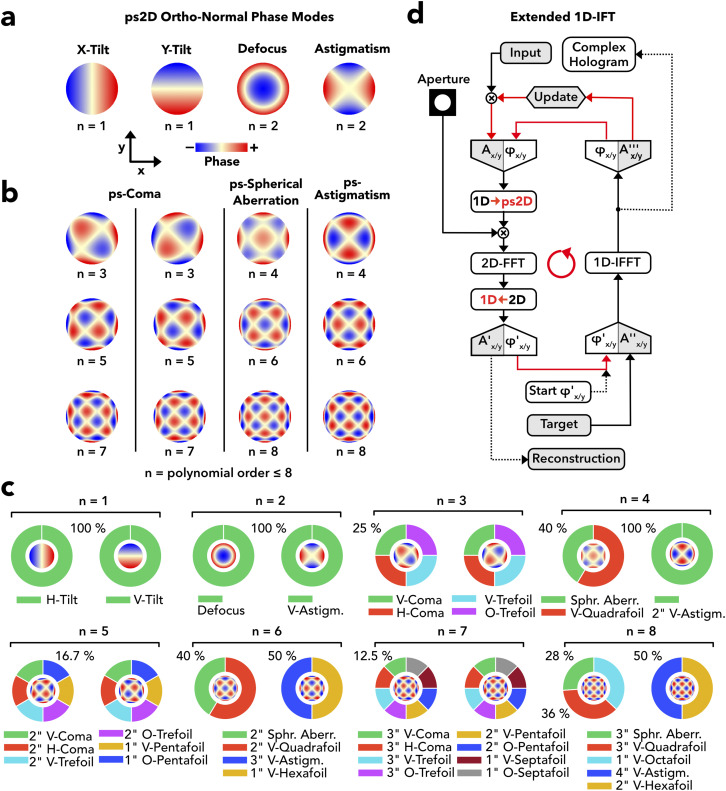
Pseudo-2D spatial light modulation (a) Ortho-normal phase functions of first and second polynomial order and obeying ps2d symmetry consistent with a biaxial modulation performed in a crossed AOD pair. (b) Same as panel (a) from third to eighth order forming the ps2D equivalents to coma, spherical aberration, and vertical astigmatism. (c) Donut plots of the decomposition of the functions in panels (a) and (b) into 2D Zernike polynomials. (d) Flow chart of the extended IFT algorithm for a retrieval of complex 1D holograms, separately for each axis (x and y), under the constraint of a wanted target and accounting for the effect of light diffraction by a circular system aperture.

While single AODs perform phase and amplitude modulation in 1D, common AOD-based systems extend their range beyond 1D by combining two AODs with their active axes oriented orthogonally in a crossed configuration [supplementary material Fig. 5(a)] permitting, for instance, random-access 2D light steering.^50,51^ However, whereas AODs may shape a 1D wavefront into practically any mathematical function, including discontinuities up to the optical resolution limit [Fig. 1(c)], the 1D-outputs superpose in the crossed configuration to yield a 2D wavefront $\varphi \left(x,y\right)=\varphi_{x}\left(x\right)+\varphi_{y}\left(y\right)$, with *φ*_*x*_ and *φ*_*y*_, the wavefronts formed by the X-AOD and the Y-AOD, respectively, and x and y, the coordinates along the AOD active axes. We shall refer to the function space of 1D-separable functions as pseudo-2D (ps2D). To characterize this space, we constructed a basis of ps2D polynomials on the unit circle for direct comparison with 2D Zernike functions^52^ (see Methods). According to this analysis, the ps2D space is entirely spanned by a set of first and second order functions, identical to the Zernike modes of tilt, defocus, and vertical astigmatism [Fig. 4(a)], together with their higher order equivalents forming the groups of pseudo(ps)-coma for uneven orders and pseudo(ps)-spherical aberration and pseudo(ps)-astigmatism for even orders [Fig. 4(b)]. From the third polynomial order onwards, the modes differ from their Zernike counterparts, with the exception of second order ps-astigmatism (fourth polynomial order) by integrating an increasing number of foil modes [Fig. 4(c)] to compensate for degrees of azimuthal modulation unavailable in a biaxial system. Zernike components are not shared between AOD modes of unequal order, which proves them to be orthogonal. The ps2D space counts 2n + 1 orthogonal polynomials up to the nth order vs n(n + 3)/2 of the full 2D Zernike space. Thus, ps2D symmetry restriction impacts high order modulations more than lower order modulations.

The same constraints must apply to holography. In particular, they define the 2D structure of valid IFT targets. A ps2D input field will transform into an output amplitude field of $A^{\prime }\left(x,y\right)=A_{x}^{\prime }\left(x\right)\cdot A_{y}^{\prime }\left(y\right)$ with $A_{s}^{\prime }\left(s\right):=\left|F\left\{e^{i\varphi_{s}\left(s\right)}\right\}\right|$, with s designating x or y andF, the Fourier transform. Hence, the output equals the spatial Cartesian product of two 1D amplitude fields, which are the reconstructions of 1D holograms, *φ*_*x*_ and *φ*_*y*_. In consequence, valid 2D targets have a rectangular shape and translational symmetry along active axes. Importantly, IFT searches for ps2D-CGHs reduce to the task of searching for 1D-CGHs for each axis separately by 1D-IFT. In this way, however, IFT fails to account for light diffraction by circular system apertures. Since the 1D-IFT models the optical aperture boundary as a slit, orthogonal superposition results in ps2D holograms optimized for square apertures. Given the importance of circular symmetry in instrumental optics, we extended 1D-IFT to include a circular diffraction boundary [Fig. 4(d)]. In extended 1D-IFT, the 1D input field is temporarily expanded into a ps2D field to impose the boundary and the 1D field is subsequently recovered from the midline of the 2D output field after 2D Fourier transformation [see Fig. 4(d); the supplementary material, Fig. 5(b); and Methods]. Accounting for the aperture effect on each of the two 1D light fields in separate IFTs, rather than by combining the two fields into a single 2D field, procures fast and data-efficient generation of ps2D holograms (see Discussion).

**FIG. 5.**
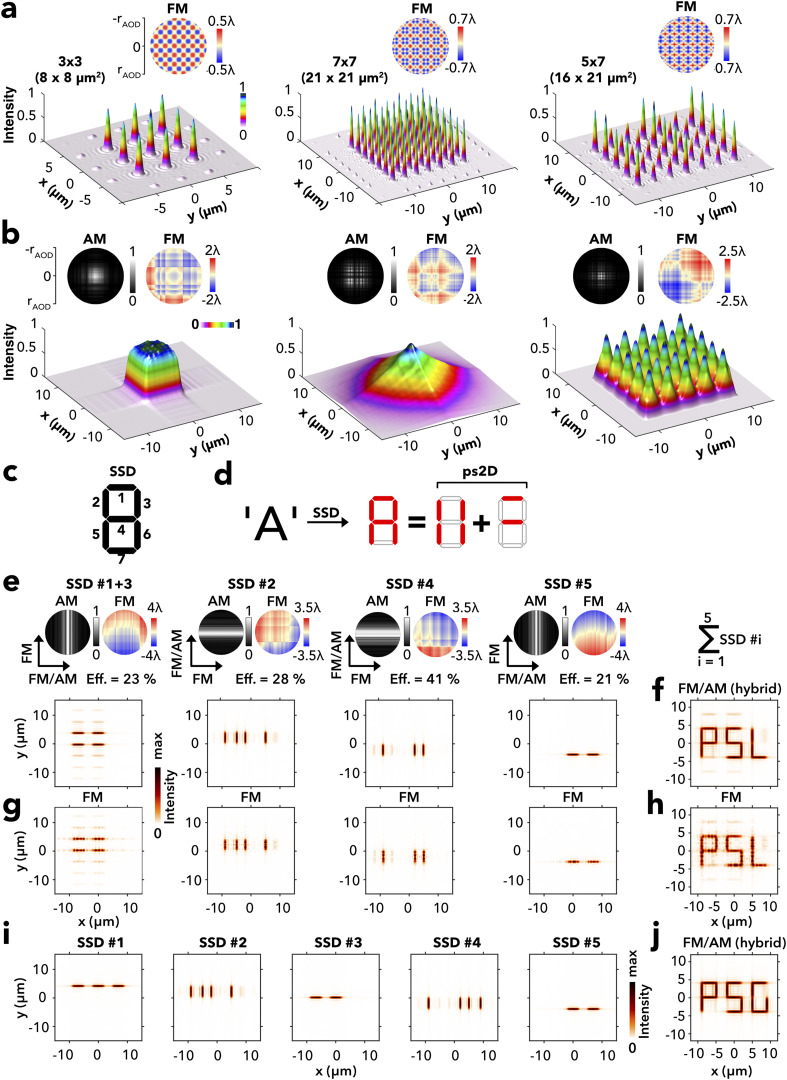
Ps2D microscopic light patterning. (a) Ps2D phase-only holograms (FM; *top*) reconstructing into point grids (*below*) of 3 × 3 (*left*), 7 × 7 (*middle*), and a 5 × 7 (*right*) diffraction-limited spots. The axial modulations correspond to the 1D targets shown in Fig. 2(c), with the 3 × 3 and 7 × 7 grids receiving identical modulation on both axes (x and y), while the 5 × 7 patters combines a five-point [x axis; second pattern shown in Fig. (2c)] and a seven-point [y axis; last pattern shown in Fig. 2(c)] target. (b) Complex holograms (*top*) and 2D reconstruction (*below*) corresponding to the targets shown in Fig. 3(f) applied simultaneously to the x axis and y axis. (c) Seven-segment display (SSD). (d) SSD representation of the letter “A” by combining segments into groups of ps2D symmetry. (e) Writing a three-letter acronym through the serial generation of SSD meta segments (Nos. 1–5; Nos. 1 and 3 merged) with complex holograms in the FM/AM hybrid mode (*top*) with their reconstruction (*below*). Power efficiency of each hologram is given in percentage of the input power (Eff.), assuming a Gaussian input beam. (f) Superposition of segments in panel (e) displaying the acronym “PSL,” for “Paris Sciences & Letters.” (g) Reconstructions of the same targets as in panel (e) using phase-only holograms (FM). (h) Sum of meta segments in panel (g). (i) Series of reconstructed SSD meta segments (Nos. 1–5), same targets as for panel (e), but changing the “L” letter to “G.” (j) Sum of meta segments in panel (i).

As anticipated, ps2D patterns inherit their properties from their constituting 1D holograms. Ps2D patterns derived from discrete point targets [Fig. 2(a)], by imposing the same target to both axes, were well reconstructed from phase-only holograms [Fig. 5(a)], while target side lobes were cancelled by adding an amplitude mask, regardless of whether the mask was delivered by post-IFT apodization or complex IFT [supplementary material Fig. 6(a)]. Ps2D patterns obtained from dense 1D targets [same as in Figs. 3(b) and 3(f)] supplied to both axes, on the other hand, required complex holography [Fig. 5(b)] as phase-only holograms expectedly rendered objects with intense speckle noise [supplementary material Figs. 6(b) and 6(c)]. At the same time, extended IFT [Fig. 4(d)] provided for better reconstruction [Fig. 5(b)] than simple IFT [supplementary material Fig. 6(d)], although with a lower margin than complex vs phase-only holography [supplementary material Fig. 6(c)]. To test for the role of the reconstruction aperture directly, we compared 2D reconstructions of simple IFT-generated holograms with either a circular [supplementary material Fig. 6(d)] or a square aperture [supplementary material Fig. 6(e)] of equal size. Reconstruction with square aperture indeed improved the rendering [supplementary material Fig. 6(f)] to a level matching the extended IFT [supplementary material Fig. 6(c)].

Finally, we explored the possibility to approximate complex light shapes beyond the competence of ps2D holography through serial holography of ps2D-compatible geometric elements. As an example, we simulated a holographic microscale version of a vintage seven-segment display^53^ [SSD; Fig. 5(c)]. In seven-segment displays (SSDs), alphanumeric text is displayed by activating groups of line segments to represent the text. Serial holography regroups the segments into ps2D-compatible targets [Fig. 5(d)] and stores each in separate holograms. To represent text across multiple SSDs, we defined five meta segments (SSD Nos. 1–5) by grouping together segments as follows: SSD No. 1 (segment 1), SSD No. 2 (segments 2 and 3), SSD No. 3 (segments 4), SSD No. 4 (segments 5 and 6), and SSD No. 5 (segment 7). In the example given [Fig. 5(e)], SSD No. 1 and SSD No. 3 were merged by recognizing their y-translation symmetry, which allows us to store them in a single hologram. The superposition of SSD reconstructions [Fig. 5(e), *bottom row*] displays the acronym of “Paris Sciences & Letters” [PSL; Fig. 5(f)]. The SSDs [Fig. 5(e), *upper row*] are designed as ps2D hybrids with one axis using the complex mode (FM/AM) and the orthogonal axis phase-only mode (FM). Hybrid SSDs provided superior reconstruction than biaxial phase-only SSDs with a visibly better line rendering [Figs. 5(g) and 5(h)]. However, the hybrid reconstructions are still prone to low intensity second order ghosts by FM modulation, mostly visible in the vertical direction of the merged SSDs Nos. 2 and 3 [Figs. 5(e) and 5(g)]. Apodizing these axes successfully removed the ghosts [supplementary material Figs. 7(a)–7(c)], albeit at reduced power efficiency (values given in the figure), in agreement with our earlier finding [Fig. 2(b)]. Changing the letter “L” against the letter “G,” for example, removes the SSD Nos. 2/3 degeneracy to yield five holograms [Fig. 5(i)] reconstructing “Paris Saint Germain” (PSG). Five is also the maximum number of holograms needed to store any text of reasonable length compatible with the SSD alphanumeric code [supplementary material Fig. 7(d)–7(f)].

## EXPERIMENTAL IMPLEMENTATION

**FIG. 6.**
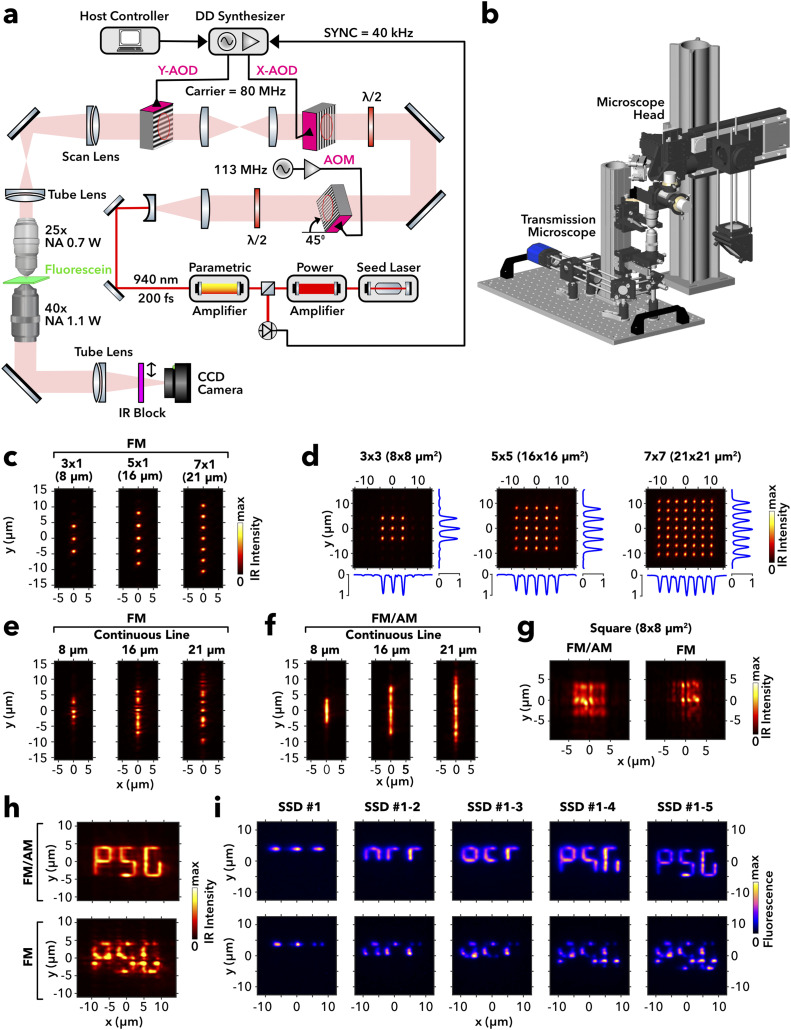
Implementation of acousto-optic holography in a two-photon microscope. (a) Optical setup featuring a parametrically amplified laser delivering 200 femtosecond pulses at 40 kHz to two synchronized AODs in crossed configuration for ps2D optical phase and amplitude modulation after dispersion compensation performed by a 45°-mounted AOM and employing two reversed objectives to reconstruct holograms and image the reconstructions of infrared (IR; 940 nm) or green fluorescence light (thin fluorescein layer) onto a CCD camera. (b) 3D model of the microscope head showing in the center the two objectives with shared focal planes. (c) False color images of the focal light patterns by phase-only (FM) modulation applied to the y axis through the Y-AOD in the absence of modulation in x direction for three (8 *µ*m length; *right*), five (16 *µ*m; *middle*), and seven (21 *µ*m; *right*) point targets. (d) Images of the 2D patterns when applying the same modulation as in panel (c) to both AODs together with maximum projections in x and y directions (blue). (e) Images of the pattern obtained in the phase-only (FM) mode for continuous lines of 8, 16, and 21 *µ*m length (*from left to right*) as targets. (f) Same as in panel (e), but by complex holography (FM/AM) at full gain (*g*_*AM*_ = 1). (g) Measured square pattern by combined modulation for 8 *µ*m lines in x and y directions (panel e; *left*) using the FM/AM (*left*) and FM *right*) mode. (h) Writing a three-letter acronym through serial generation of five SSD segments in FM/AM (*top*) and FM (*bottom*) modes. (i) False-color images of green fluorescence emission by two-photon absorption of IR light in a thin fluorescein layer with serial writing of SSD segments one to five in FM/AM (*top row*) and FM mode (*bottom row*). The images sequences show the accumulated fluorescence during a serial writing of the acronym employing one laser pulse per segment.

To validate the numerical reconstructions, we performed experimental reconstructions of several CGHs by imaging their intensity distribution in the focal plane of an AOD-empowered random-access microscope [supplementary material, Figs. 8(a) and 8(b)] via an inverted transmission microscope onto an image detector [Fig. 6(a) and Methods]. First, patterns composed of isolated single points arranged on a line [Fig. 6(c)] or on a square lattice [Fig. 6(d)] were well reconstructed from phase-only holograms (FM) with all points sharing nearly equal intensity. In contrast, continuous lines, representing dense targets, reveal marked random intensity inhomogeneity in the FM mode, reminiscent of random speckle [Fig. 6(e)]. Complex hologram reconstruction (FM/AM) visibly homogenized the intensity distribution along lines [Fig. 6(f)] and across square targets [Fig. 6(g)]. Next, we imaged the focal light intensity, while the scan engine looped through a sequence of five holograms, each representing one SSD meta-segment of a three-letter acronym. Since each hologram was reconstructed with a single pulse of a 40 kHz infrared laser, the sequence was completed within 125 *µ*s. In particular, this is much faster than the camera frame cycle, which is why the camera images show the intensity of all meta segments combined [Fig. 6(h)]. Again, the image was neat in hybrid [Fig. 6(h), *top*] and visibly disturbed in the phase-only [Fig. 6(h), *bottom*] mode. We also imaged the light emission by two-photon excitation of a thin fluorescent layer (600 nm thick color plastic slide) placed in the focal plane of the microscope. Because of the second order non-linearity of two-photon excitation, we expected the fluorescence image to enhance the inhomogeneities present in the excitation pattern. In the phase-only case, the reconstruction was indeed no more readable, in contrast to the hybrid mode [Fig. 6(i)], in agreement with the numerical reconstructions of same CGHs (supplementary material, Fig. 9).

## CONCLUSIONS

Acousto-optic spatial light modulation was recently shown to allow the projection of complex ps2D holograms comprising phase and amplitude information.^17^ To render AO holography practically useful, we report tailored methods to computer-generate ps2D holograms for acousto-optic devices. Building upon established methods in digital holography, we described the retrieval of ps2D holograms depending on *a priori* defined target patterns. In particular, we present regularizations for complex holography in the (FM/AM) mode, together with improvements in previous IFT algorithms for phase-only holograms. We demonstrated hologram retrieval with point grids and line patterns as chosen targets and validated retrieved CGHs by numerical and experimental reconstruction. Although our simulations used a specific set of instrumental parameters, these parameters easily generalize to other configurations.

Holographic reconstructions are plagued by speckle noise because diffuse illumination with coherent light creates a random-phase field in the object plane. As a consequence, reconstructed overlapping phase fields cause speckle-type amplitude noise by random interference.^43^ Speckle noise may be suppressed by averaging multiple reconstructions subject to independent noise^16,54^ or by summing reconstructions of sparse non-overlapping pixel groups,^55,56^ for instance. However, these methods sacrifice reconstruction speed. Averaging can be avoided altogether by making use of the holographic amplitude provided in the complex CGH. The reconstruction of complex CGHs has been demonstrated with two cascaded phase-only SLM, as well as single SLMs.^24,57,58^ In the cascaded SLM configuration, the first SLM projects an amplitude pattern onto a second SLM for the latter to add the appropriate phase.^23,55^ While the configuration provides 2D control of the complex light field, the configuration is liable to registration issues and diffraction ghosts. The single-SLM configuration, on the other hand, applies high spatial frequency modulation of the phase in combination with a spatial Fourier filter such that high frequency components are blocked by the filter, while low frequency components pass through with their amplitude modulated complementary to the power carried by the blocked high frequency components.^24,57–59^ While the method has the advantage of employing a single modulator, the spatial resolution is lower than the SLM pixel resolution and liable to high alignment sensitivity. In a somewhat related approach, phase and amplitude may be conferred to non-overlapping randomized pixel groups with one group tasked to create the amplitude trough random diffraction of excess power,^25,60^ at the disadvantage of low resolution, possibly elevated background, and a likely need for temporal averaging. AODs offer a simple and robust alternative in ps2D geometry at a superior update rate and high damage threshold. AODs, being analog devices, provide an ultra-stable FM/AM performance by using the same carrier for generating the FM and AM side bands, which precludes temporal or spatial aliasing between amplitude and phase masks and thus renders them robust, for instance, against drifts in ambient conditions and optical alignment. Given the unavoidable power loss in the FM/AM AOD mode [Fig. 3(e)], it is notable that the expected loss ends up not too far from the measured losses in cascaded SLMs, where power loss is caused by scattering and fill factor.^23^ Acousto-optic holographic amplitude modulation, on the other hand, was demonstrated only recently^17^ and requires the synchronous mode^30^ [Figs. 1(a) and 1(b)] to prevent phase drift and intensity flicker linked to acoustic wave propagation. Standard AO systems, however, are operating in an asynchronous mode^34,61^ for general compatibility with quasi-continuous lasers and, hence, are not susceptible to FM/AM holography. Similarly, the proposed AO holographic image projection uses the FM mode and relies on time averaging to battle speckle noise, which currently limits them to video rate.^16^ Therefore, FM/AM offers the opportunity to accelerate ps2D dynamic holography without immanently boosting the noise.

Regularization of IFT offers convenient ways to tailor ps2D-CGHs to match application-defined targets based on the paradigm of pulse-to-pulse pattern update and pulse-wise selection of modulation mode, either FM, FM/AM, or hybrid. This goes in hand with the alleviated CGH retrieval because of orders of magnitude lower computational FFT load, leaner digital data and, as a result, faster execution, compared to unconstrained 2D holography (see Methods). AO holography appears particularly suited to applications being either intrinsically compatible with a ps2D geometry or firmly prioritizing speed over unconstrained 2D modulation. Indeed, AODs proved fast enough for active pulse-to-pulse wavefront shaping of amplified laser pulses up to 150 kHz in anisotropic^30^ and up to 1.6 MHz in isotropic^62^ Bragg diffraction. AODs also offer large acoustic bandwidths to drive continuous phase modulation up to 10^3^ phase periods without 2π reset. Such depths of modulation largely outperform MEMS-SLMs and help avoid diffraction ghosts, known from 2π- or 4π-limited modulators, such as liquid–crystal SLMs.^63^ Discontinuities of the holographic phase other than 2π, eminent to holograms of certain targets, in particular dense patterns, are reproduced in AODs up to the limit of the allocated acoustic bandwidth analogous to the impact of pixel resolution in other types of SLMs. At the same time, as the present industrial fabrication allows us to grow and cut high-quality AOD crystals to sizes comparable to the entrance aperture of common high NA objectives, they may ultimately reach comparable diffraction-limited optical resolution, at least for acoustic frequencies close to the carrier wave. Finally, ps2D-CGHs are void of spiral phase vortices since full vortices do not exist in one dimension. Vortices are known to be detrimental to 2D holography,^64^ cause local IFT stagnation,^65^ and are impossible to unwrap. The absence of vortex-type phase singularities, therefore, helps minimize phase error in ps2D holography. On the flip side, the approach is compatible only with pulsed lasers of comparatively low repetition rate and viable light patterns are constrained to biaxial symmetry. AOD light modulation also requires careful compensation of AOD angular chromatic dispersion^66–68^ and a judicious usage of the available AOD bandwidth (see Methods).

Acousto-optic holography is a very recent development, and despite the maturity of AO device technology established decades ago,^28^ new applications continue to emerge. AO light patterning recently enabled new experimental paradigms of light control in random-access recordings of biological activity *in vivo*,^17,69,70^ building upon previous AO systems for fast 3D access,^71–73^ including real-time compensation of target motion.^74^ These and related applications commonly rely on digital holography as the means to retrieve and model holographic representations of structured light. With these methods at hand, AO holography supplies a valuable tool for innovative applications in optical bioscience and possibly other areas, such as display technology, material processing, or metrology.

## SUPPLEMENTARY MATERIAL

The supplementary material contains nine supplementary figures, cited in the test as supplementary material Figs. 1–9, and a methods description, cited as Methods.

## Data Availability

The data that support the findings of this study are openly available at https://github.com/walther007/3D-CASH.
